# Practical Aids in Medical and Surgical Diagnosis

**Published:** 1910-06

**Authors:** Lewis M. Gaines

**Affiliations:** Atlanta, Ga.; Professor of Physiology Atlanta School of Medicine; Associate Medical Director Peachtree Heights Sanatorium; Physician to Presbyterian Hospital


### Journal-Record of Medicine

Successor to Atlanta Medical and Surgical Journal, Established 1855, and Southern Medical Record, Established 1870.

OWNED BY THE ATLATNA MEDICAL JOURNAL CO.

Publishsd Monthly

*Official Organ Fulton County Medical Society, State Examining Board, Presbyterian Hospital, Atlanta, Birmingham and Atlantic Railroad Surgeons' Association, Chattahoochee Valley Medical and Surgical Association, Etc.*

EDGAR G. BALLENGER, M. D., Editor.
BERNARD WOLFF, M. D., Supervising Editor.
A. W. STIRLING, M. D., C. M., D. P. H., J. S. HURT, B. Ph., M, D,
GEO. M. NILES, M. D., W. J. LOVE, M. D., (Ala.); Associate Editors.
E. W. ALLEN, Business Manager.

COLLABORATORS

DR. W. F. WESTMORLAND, General Surgery.
F. W. McRAE, M. D., Abdominal Surgery.
H. F. HARRIS, M. D., Pathology and Bacteriology.
E. B. BLOCK, M. D., Diseases of the Nervous System.
MICHEL HOKE, M. D., Orthopedic Surgery.
CYRUS W. STRICKLER, M. D., Legal Medicine and Medical Legislation.
E. C. DAVIS, A. B., M. D., Obstetrics.
E. G. JONES, A. B., M. D., Gynecology.
R. T. DORSEY, Jr., B. S. M. D., Medicine.
L. M. GAINES, A. B., M. D., Internal Medicine.
J. N. LeCONTE, M. D., Diseases of the Stomach and Intestines
L. B. CLARKE, M. D., Pediatrics.
EDGAR PAULIN, M. D., Opsonic Medicine.
R. R. DALY, M. D., Medical Society.
A. W. STIRLING, M. D., Etc., Diseases of the Eye, Ear, Nose and Throat.
BERNARD WOLFF, M. D., Diseases of the Skin.
E. G. BALLENGER, M. D., Diseases of the Genito Urinary Organs.

VOL. LVI. JUNE, 1910 No. 3.

INDEX THROUGH

### PRACTICAL AIDS IN MEDICAL AND SURGICAL DIAGNOSIS.

BY LEWIS M. GAINES, A. B., M. D., ATLANTA, GA.

*Professor of Physiology Atlanta School of Medicine; Associate Medical Director Peachtree Heights Sanatorium; Physician to Presbyterian Hospital.*

### FOREWORD.

Has every reader of these lines considered that the marvelous advances of the science of medicine in the past few decades have imposed upon him a tremendous responsibility? Are physicians giving to their patients the full benefit of scientific methods in the diagnosis of chronic or puzzling maladies?

Much—possibly enough—has been said concerning the importance of correct diagnosis in the treatment of disease, but are physicians making the use they should of those methods that lead to correct diagnoses?

Practically speaking, it is impossible for the general practitioner to utilize all, or even a large proportion of these methods. Many of them require far more time than he has to give, many of them require delicate and expensive apparatus, that he might not be justified in obtaining, and finally, many of these methods require a degree of skill that is the reward of such constant practice as the general practitioner is often unable to devote to so limited a field. Beside all these considerations, there never has been a time when more new and valuable methods are constantly being brought out by the scientific workers of the world. To keep abreast of these new methods requires such a degree of constant study and watchfulness as no one would or could expect the general practitioner to give.

It would appear, therefore, from the above conclusions, that children, there are two important factors: First, the inherited it would be advantageous for physicians generally to be reminded of just what methods are of the greatest help to them in their daily work, in just what disorders they should employ specific methods, and finally, if they themselves for any of the reasons mentioned above are not in a position to make such examinations or use such methods as are required, they would still be able to take advantage of these helps by calling upon those who do devote their time and energies to such work.

It shall be my attempt in a series of papers to take up each month a number of different methods of diagnosis, and show wherein they are of practical value in the diagnosis and treatment of disease.

### I.

### The Practical Value of Blood Examinations.

The present methods of blood examination as a means of diagnosis and prognosis have been in vogue a comparatively short time. This fact explains in large part the infrequent employment of this valuable aid in observing diseased conditions by some, as well as the exaggerated claims of the importance of blood examinations made by others. These considerations have suggested the utility of an inquiry into the real practical value to the physician and to the surgeon of having blood examinations done on the cases each encounters in his daily work, for as has been remarked, the value of a blood examination is measured by the practical use which may be made of it and not by any interesting but useless information it may throw upon the case. It has seemed to me that to busy practitioners who have not closely investigated the subject of blood work, questions such as these might suggest themselves: (1) Just what is meant by a blood examination? (2) In what class of cases will a blood examination be of value? (3) How will a blood examination help me in treating these cases? (4) Will a blood examination help me in giving a prognosis?

This paper represents an endeavor to answer these questions. (1) What is meant by a blood examination? This term as ordinarily employed refers to the following proceedures:

Enumeration of the number of red blood corpuscles and of the leucocytes per cubic millimeter of blood, estimation of the percentage amount of hæmoglobin, an examination of the fresh unstained blood and of the stained smear for malarial parasites and for the presence of various abnormalities, and finally by enumeration, ascertaining the percentage number of the different kinds of leucocytes present.

This last preceedure is known as the differential count.

Frequently any one of the above mentioned proceedures is termed a blood examination, whereas all of them are embraced under the name of a *complete* blood examination.

In its widest sense a blood examination would include a bacteriological examination to ascertain the presence of micro-organisms in the circulation, or the existence of agglutinating phenomena in blood sera, such as the Widal reaction, or the ascertaining of the opsonic index. With this bacteriological examination this paper is not concerned.

Furthermore there are certain other proceedures which might be termed "special examinations" which are of occasional value, such as estimation of coagulation time, and the specific gravity.

the detection of iodophilia and the presence of granulations in the red blood corpuscles.

(2) In what class of cases will a blood examination be of value?

This question may be discussed with advantage from the standpoint, first of the surgeon, second, of the physician.

From a surgical standpoint, examination of the blood is a valuable means of diagnosis in comparatively few instances, but in those instances they are almost indispensable. I refer to the leukaemias, Hodgkin's disease, and the confusion of typhoid fever with inflammatory conditions.

It is an interesting fact that a large percentage of the cases of spleno-myelogenous leukaemia referred to the Johns Hopkins Hospital were sent as surgical cases for operation upon an abdominal tumor. A glance at the blood readily reveals the nature of the tumor but in no other way can the correct diagnosis be reached.

In cases of lymphatic leukaemia with glandular enlargement the mistake is frequently made of confusing them with glandular enlargement due to other causes. A case recently referred to me by Dr. M. B. Hutchins, of Atlanta, for blood examination had been treated locally for a year with no reference to the true condition.

If we take 6,000 leucocytes as a normal count, typhoid fever uncomplicated will invariably cause a dimunition in the number, whereas acute inflammatory conditions will invariably show an increased count if the resistance is good. This, at times, is a very valuable aid to diagnosis. I have seen the abdomen opened for appendicitis in typhoid fever, and I have known osteomyelitis to be regarded as typhoid fever. In either case a blood examination might have solved the problem.

The most frequent answer, however, that the surgeon demands of the blood examination is whether or not to operate on a given case the diagnosis of which is clear. In the majority of cases the results of blood examination have no particular influence on the question of operative interference. The surgeon is guided more by the clinical symptoms and disregards the blood examination, and may usually do so with safety.

Before proceeding further let us inquire the effect of inflammation on the blood picture. If there is good resistance on the part of the body, a leucocytosis more or less marked results varying from 15,000 to 35,000, occasionally higher. On the other hand, if the body resistance is poor there may be no change in the count from normal or there may be even a dimunition of the count, a leucopaenia. But there is another change of even more importance; and that is the alteration of the differential count. In cases where the polymorphonuclear leucocytes are relatively increased above the normal a purulent or gangrenous termination of the inflammation is indicated, while if the percentage of these cells is normal or below, these events are excluded at the time the examination is made.

A few illustrative cases quoted from Fowler, admirably illustrate the point in question.

(1) Young woman with serious otitis media. Owing to extreme pain and other clinical symptoms acute mastoid disease was suspected. Leucocyte count 28,400. Polymorphonuclears 59.7%. Clinical picture and leucocytosis would have indicated operation, but the normal polymorphonuclears percentage led the aurist to wait and a prompt recovery without purulent exudate made operation unnecessary.

(2) Young woman in apparently good condition. Severe pelvic cellulitis with vague manifestation of abscess formation. Leucocyte count 7200, polymorphonuclears, 87%. This high percentage indicated the necessity for immediate operation which revealed a large collection of pus. Operation followed by recovery.

(3) Rather feeble elderly lady with typical clinical evidence of appendicitis. The surgeon advised immediate operation, the physician advised waiting. Leucocytes, 13,200; polymorphonuclears, 82.4%. Owing to the high polymorphonuclears percentage the operation was done and the surgeon found a perforated, gangrenous appendix with general spreading peritonitis.

The conclusions, therefore, to be drawn are that the leucocyte count alone is an evidence of the extent of body resistance and bears on prognosis, whereas the polymorphonuclears percentage is an indication for or against the necessity of immediate operation according as it is high or low, and that in cases where the propriety of immediate operation is in doubt, the aid of the blood examination should be invoked.

The extent of anaemia in operative cases is at times a matter of much importance, for it is well known that patients with a high grade of anaemia stand operation badly. The extent of this anaemia should be determined by the enumeration of the red cells as well as by an estimation of the hæmoglobin. For example: A patient with hæmoglobin 30% and red count 4,000,000 will stand operation better than a case with hæmoglobin 60%, and red count 2,000,000. In other words, it is important to determine the type of anaemia present.

Finally, in cases with extreme jaundice, the determination of coagulation time of the blood may be very valuable. One case has come under my observation where death from hemorrhage followed operation upon a jaundiced patient on account of the greatly increased coagulation time which might have been shortened by proper treatment before operation.

For the physician, blood examinations are even more valuable than for the surgeon. Leukaemia and Hodgkin's disease, to which reference has already been made, can only be diagnosed in this way. Pernicious anaemia is usually overlooked without the blood examination and frequently regarded as jaundice or malaria, for which they are treated with no benefit. Although many cases of malaria are recognized and successfully treated without reference to the blood, the disease is best recognized by blood examination, especially those a typical forms with no paroxysms. As proof of this statement consider the long list of diseases for which malaria has been mistaken: typhoid fever, meningitis, uraemic coma, pernicious anaemia, tuberculosis, dysentery. A mistake in cases of pernicious malaria means misdirected treatment and unnecessary death.

Furthermore, it should be remembered that a negative finding is of as much importance as a positive finding. The fact that no abnormal condition is present does not indicate that the blood examination was a useless proceedure, but that certain suspected pathological conditions can be excluded with certainty. It should also be remembered that in many cases, to be of real value, a number of blood examinations should be made just as truly as the temperature or the pulse is taken many times. In many diseases it is the blood curve that furnishes more valuable information than an isolated count.

In general a blood examination is of value to a physician in all cases of pronounced anaemia, of jaundice, of suspected malaria, and in all cases where the puzzling nature of the symptoms render the diagnosis uncertain.

(3) How will a blood examination aid in the treatment of a case?

No one can deny that the most important thing in the treatment of any condition is a correct diagnosis. Granted, however, that the diagnosis has been successfully made, there are certain diseases whose progress can best be followed by means of the blood, just as others can best be studied by means of the temperature. This is particularly true of the primary anaemias: the leukaemias and pernicious anaemia, and in many cases of secondary anaemia. The most reliable index of the effect of treatment can be obtained only by a careful blood examination made at regular intervals. While the general appearance of a patient is often fairly accurate, one will occasionally be deceived. I have seen patients of very rosy hue but with a low red count, whereas others with a pallid cast of countenance show a normal count.

In typhoid cases suspected of perforation with steadily rising leucocyte count, particularly of the polymorphonuclears, is sometimes of the greatest value in determining the psychological moment for operative interference.

(4) Finally, the result of a blood examination is of great value in the prognosis of certain cases.

From the patient's standpoint, this is most important. His most insistent inquiry is, "Will I get better?" In all cases where the blood count is of any value, it is of value in prognosis. If your anaemic patient suffers from pernicious anaemia, how different the outlook from that of another suffering from a secondary form. The pneumonia with a normal leucocyte count has a far slimmer chance than if it were very high. The malaria case infected with the tertian organism may be cured with greater ease than the one with the aestivo autumnal. These only are examples of the practical aid in prognosis rendered by blood examination in properly selected cases.

In conclusion, it may be said that while the profession should be conservative in attaching importance to any special method of investigation, still the practical value as well as the limitations of blood work should be more generally and accurately recognized. By so doing it is certain that in many instances mistakes would be rectified, therapy would be rendered more certain, more definite prognoses would be given, and most important of all, more human lives would be saved.

(The second paper of the series will be on the practical value of urinary examinations).

